# Mr-lac3 and Mr-lcc2 in *Metarhizium robertsii* Regulate Conidiation and Maturation, Enhancing Tolerance to Abiotic Stresses and Pathogenicity

**DOI:** 10.3390/jof11030176

**Published:** 2025-02-22

**Authors:** Qiaoyun Wu, Yingying Ye, Yiran Liu, Yufan He, Xing Li, Siqi Yang, Tongtong Xu, Xiufang Hu, Guohong Zeng

**Affiliations:** Zhejiang Province Key Laboratory of Plant Secondary Metabolism and Regulation, College of Life Science and Medicine, Zhejiang Sci-Tech University, Hangzhou 310018, China; wqy18871931436@163.com (Q.W.); yeyy18861985920@163.com (Y.Y.); 18630053015@163.com (Y.L.); 15088602089@163.com (Y.H.); 19012728985@163.com (X.L.); yangss118@163.com (S.Y.); xdy1041@163.com (T.X.); huxiuf@sina.com (X.H.)

**Keywords:** *Metarhizium robertsii*, laccases, Mr-lac3, Mr-lcc2, conidiation, conidial maturation, abiotic stresses, pathogenicity

## Abstract

As a type of multicopper oxidase, laccases play multiple biological roles in entomopathogenic fungi, enhancing their survival, development, and pathogenicity. However, the mechanisms by which laccases operate in these fungi remain under-researched. In this study, we identified two laccase-encoding genes, *Mr-lac3* and *Mr-lcc2*, from *Metarhizium robertsii*, both of which are highly expressed during conidiation. Knocking out *Mr-lac3* and *Mr-lcc2* resulted in a significant increase in the conidial yields of *M. robertsii*. Furthermore, the relative expression levels of upstream regulators associated with the conidiation pathway were markedly up-regulated in *ΔMr-lac3* and *ΔMr-lcc2* compared to the wild-type strain during conidiation, indicating that *Mr-lac3* and *Mr-lcc2* negatively regulate conidia formation. qRT-PCR analyses revealed that *Mr-lac3* and *Mr-lcc2* are regulated by the pigment synthesis gene cluster, including *Mr-Pks1*, *Mr-EthD*, and *Mlac1*, and they also provide feedback regulation to jointly control pigment synthesis. Additionally, *ΔMr-lac3* and *ΔMr-lcc2* significantly reduced the trehalose content in conidia and increased the sensitivity to cell wall-perturbing agents, such as Congo red and guaiacol, which led to a marked decrease in tolerance to abiotic stresses. In conclusion, the laccases Mr-lac3 and Mr-lcc2 negatively regulate conidia formation while positively regulating conidial maturation, thereby enhancing tolerance to abiotic stresses and pathogenicity.

## 1. Introduction

Multicopper oxidases are a class of enzymes that include laccases and copper oxidases, which catalyze the oxidation of a variety of aromatic and inorganic substances coupled with the reduction of O_2_ molecules to H_2_O [1]. In recent years, the structures of many fungal laccases have been solved by X-ray crystallography. The amino acid sequences of all multicopper oxidases contain a 10–20 kDa cupredoxin-like domain [2].

In fungi, laccases are involved in many important biological processes, such as lignocellulose degradation, defense, pathogenesis, pigmentation, sporulation, and others. Fungal laccases, especially those from white-rot species, play key roles in lignocellulose biodegradation and thus contribute to carbon recycling in the biosphere [3]. In addition, the secretion of laccases is induced when fungi are stressed by factors such as microorganisms, xenobiotics, metals, toxins, and biologically active compounds. Recently, Lakshmanan and Sadasivan reported that *Trichoderma viride* laccase plays a crucial role in defense against antagonistic organisms [4].

Similarly, *Botrytis cinerea* BcLcc2 laccase was found to be involved in resistance against antibiotics such as 2,4-diacetylphloroglucinol (2,4-DAPG) with tannic acid as the redox mediator [5]. In many pathogenic fungi, melanin pigments found in conidia or within cell walls are antimicrobial and act as virulence factors, while fungal laccases are the key enzymes of melanin metabolism. In *Cryptococcus neoformans* ATCC 3483 (phylum Basidiomycota, order Tremellales, and family Cryptococcaceae), CNLAC1 laccase acts as the main diphenol oxidase involved in melanin biosynthesis and is necessary for the pathogenic ability to infect humans [6,7]. *Talaromyces marneffei* is an emerging opportunistic pathogen associated with HIV infection, and its LacA, LacB, LacC, and PbrB laccases promote pathogenic resistance to innate host defenses [8]. *Colletotrichum gloeosporioides* LAC1 laccase is crucial for the virulence of plant pathogenic fungi on mango leaves and fruits. *Metarhizium rileyi Mrlac1* laccase is involved in the complete virulence to insect hosts and is required for good adaptability in fields [9]. Additionally, it plays a role in various developmental processes, including mycelial growth, differentiation, conidiation, melanin biosynthesis, pigmentation, appressorium formation, the secretion of extracellular hydrolytic enzymes, and the utilization of exogenous nutrients [10].

*Metarhizium robertsii* (phylum Ascomycota, order Hypocreales, and family Clavicipitaceae) is an ascomycete with a cosmopolitan distribution in diverse ecological niches, including strains that are saprophytes, plant symbionts, and insect pathogens [11]. Because of its multiple lifestyles, *M. robertsii* has been developed into microbial preparations that can be used as fungal insecticides and bio-fertilizers, which reduces the use of chemical pesticides and fertilizers [12]. *M. robertsii* has strong tolerance to abiotic stresses, including cold, heat, UV radiation, and oxidation, enabling it to maintain large populations in fields [13,14,15,16]. In order to further enhance the efficiency of mycoinsecticides, detailed studies are needed on the mechanisms of pathogenesis, conidiation, and conidial stress tolerance of *M. robertsii*. Previously, Fang et al. identified the laccase gene *Mlac1* (Metarhizium laccase 1), which is a virulence determinant expressed only in infective appressoria and blastospores, contributing to their cell wall rigidity, pigmentation, and tolerance to abiotic stresses like heat shock (45 °C for 2 h) and UV-B radiation [17]. In a further study, a new pigmentation gene cluster was discovered in *M. robertsii*, encoding Mr-Pks1, Mr-EthD, and Mlac1. The conidial pigment produced by the gene cluster was found to have varying effectiveness against UV-A and UV-B radiation [18]. In previous studies, we found that *Mr-WetA*, a key component in the conserved conidiation regulatory pathway in *M. robertsii*, controls the formation and development of conidia [18]. Additionally, further RNA-Seq analysis revealed a significant down-regulation of the expression levels of 3 laccase-encoding genes [*Mlac1* (*MAA_07747*), *Mr-lac3* (*MAA_04762*), and *Mr-lcc2* (*MAA_07580*)] in *ΔMr-WetA* during conidiation. And previous studies have shown that *Mlac1* is both a virulence determinant and is required for tolerance to abiotic stresses [17]. However, the molecular mechanisms of *Mr-lac3* and *Mr-lcc2* are still unclear. Both *Mr-lac3* and *Mr-lcc2* are single-copy genes, with *Mr-lac3* having an open reading frame (ORF) of 2285 bp encoding 683 amino acid residues, and *Mr-lcc2* containing an ORF of 2265 bp encoding 686 amino acid residues. Knock-out mutants and overexpression strains were then constructed to systematically characterize their biological roles in conidiation, conidial maturation, and tolerance to abiotic stresses.

## 2. Materials and Methods

### 2.1. Fungal and Bacterial Strains

*Metarhizium robertsii* ARSEF2575 was obtained from the Agricultural Research Service Collection of Entomopathogenic Fungi. *Escherichia. coli* strain DH5α was used for plasmid construction. *Metarhizium robertsii* transformation was conducted using *Agrobacterium tumefaciens* AGL1 [19].

#### 2.1.1. Gene Deletion, Complementation, and Overexpression of *Mr-lac3* and *Mr-lcc2*

Gene disruption of *Mr-lac3* and *Mr-lcc2* based on homologous recombination was conducted as previously described [20]. The plasmid used for gene deletion was pPk2-bar-GFP [19]. To construct a complementation vector, the DNA fragment of the target gene, including the promoter region (approximately 1.5 kb upstream of the ORF), ORF, and termination region (approximately 200 bp downstream of the ORF), was amplified with PCR using high-fidelity DNA polymerase (KOD Plus Neo, Toyobo, Japan) and cloned into the plasmid pPk2-Sur-GFP [19] to produce the vector pPk2-Sur-GFP complementation, which was then ectopically transformed into the corresponding gene knock-out mutant via *A. tumefaciens* AGL1. Transformants were screened based on chlorsulfuron resistance and the presence of GFP and finally confirmed by PCR. The plasmid used for gene overexpression was pPk2-sur-GFP-Tef [11]. The coding sequences of *Mr-lac3* and *Mr-lcc2* were cloned by PCR and inserted downstream of the constitutive promoter *Ptef* in the plasmid pPk2-sur-GFP-Tef. The resulting overexpression vectors were transferred into the WT of *M. robertsii* via *A. tumefaciens* AGL1 [18]. Transformants were preliminarily screened based on chlorsulfuron resistance and the presence of GFP. Overexpression strain was confirmed by qRT-PCR. All primers used in this study are listed in Supporting Information Table S1.

#### 2.1.2. Assays of Conidial Yield and Tolerance to Abiotic Stress

Conidial yields and tolerance to abiotic stress were determined as previously described [21]. Conidiophores were observed daily beginning 2 to 5 days after inoculation. Tolerance to heat stress was assayed by measuring the germination rate of conidia in 1/2 SDY liquid medium and the growth rate of colonies on PDA (BD, Franklin Lakes, NJ, USA) at 37 °C. Tolerance to oxidative stress, hyperosmotic stress, and stresses of cell wall-disturbing agents was assayed by measuring the germination rate of conidia in 1/2 SDY liquid medium supplemented with 0.005% H_2_O_2_ (Sangon Biotech Co., Ltd., Shanghai, China) and 0.75 M KCl (Sangon Biotech Co., Ltd., Shanghai, China) and the growth rate of colonies was studied on PDA supplemented with 0.005% H_2_O_2_, 0.75 M KCl, Congo red (1.5 mg/mL)(Sangon Biotech Co., Ltd., Shanghai, China), and 0.04% guaiacol (Sangon Biotech Co., Ltd., Shanghai, China) at 26 °C. Conidial germination was observed every 2 h using an inverted microscope and colony diameter was measured every day from 3 days to 15 days post inoculation. The relative germination inhibition of a given stressor was calculated as (G_c_ − G_T_)/G_c_ [22]. Each conidial yield assay and tolerance assay was repeated three times with three replicates per repeat.

#### 2.1.3. Assays of Trehalose Content

The trehalose content was determined as described before [23]. Fifteen-day-old conidia (2 × 10^8^ conidia) were washed with ddH_2_O three times, resuspended in 2 mL ddH_2_O, and incubated at 100 °C for 20 min. The suspension was then centrifuged for 10 min at 11,000× *g*, and the supernatant containing trehalose was collected. Then, the trehalose content was measured using a commercial assay kit (Tongwei Biotechnology Co., Shanghai, China).

### 2.2. Bioassays

For assays of appressorial formation rate, 70 μL of conidial suspension (3 × 10^7^ conidia mL^−1^) was inoculated on the hydrophobic surfaces of a Petri dish with 3 mL of a 0.01% YE (Yeast Extract) (Corning, Corning, NY, USA) as previously described [21].

*Galleria mellonella* of the last instars (Huiyude Biotechnology Co., Tianjin, China) were used for bioassays. Conidial suspension (3 × 10^7^ conidia mL^−1^) was applied topically to the *Galleria mellonella* as previously described [24]. Insect mortality was recorded daily. Bioassays were repeated three times with 40 insects per repeat.

### 2.3. qRT-PCR

Fungal total RNA was extracted using TRIzol reagent (Life Technologies, Carlsbad, CA, USA). The samples were prepared as previously described [18]. Briefly, conidia (1 × 10^8^ conidia) were inoculated into 100 mL of SDY (Sabouraud dextrose medium with Yeast Extract) and incubated at 26 °C for 36 h to collect mycelia. For the sample during conidiation, 100 μL of conidial suspension (1 × 10^7^ conidia mL^−1^) was evenly spread on a PDA plate (90 mm diameter, BD, Franklin Lakes, NJ, USA) and incubated at 26 °C for 5 days. For the sample during cuticle penetration, the cuticles of *G. mellonella* larvae were surface-sterilized in potassium tetraborate. A conidial suspension (200 μL 1 × 10^7^ conidia mL^−1^) was applied to each cuticle which was then placed on a 1% water agar plate and incubated at 26 °C for 30 h.

qRT-PCR analysis was conducted as previously described [21]. Total RNA (400 ng per reaction) was reverse transcribed into cDNA using HiScript 111 RT Super Mix (Vazyme, Nanjing, China). Quantitative RT-PCR analysis was performed with Taq Pro Universal SYBR qPCR Master Mix (Vazyme, Nanjing China). Gpd and tef were used as internal standards. The relative normalized gene transcription levels were calculated using the 2^−ΔΔCt^ method [22]. All qRT-PCR assays were repeated three times with three technical replicates per repeat. All primers used in this study are listed in Table S1.

## 3. Results

### 3.1. Construction of Mr-lac3 and Mr-lcc2 Disruption, Complementation, and Overexpression Strains

To systematically characterize the functions of *Mr-lac3* and *Mr-lcc2* in *M. robertsii*, we, respectively, deleted and overexpressed the entire predicted ORF (open reading frame) of *Mr-lac3* and *Mr-lcc2*. For each gene, D1, D2, and D3 designate three independent disruption mutants, and WT is the wild-type strain. The upper panel (Yes) represent PCR conducted with the primer Bar-up and the confirmation primer CF-2 (the relative positions of all primers are shown in Figure S1A) and PCR products can be obtained only from the KO mutants; The lower panel (No) represents PCR conducted with the CF-1 primer, and CF-2 and PCR products can be obtained in the WT strain but not in the KO mutants. C1, C2, and C3 are independent complemented strains; O1, O2, and O3 are independent overexpressed strains (Figure S1).

### 3.2. The Conidiophores and Conidial Yields of Mr-lac3 and Mr-lcc2 Disruption, Overexpression, and Complementation Strains

**Figure 1:**
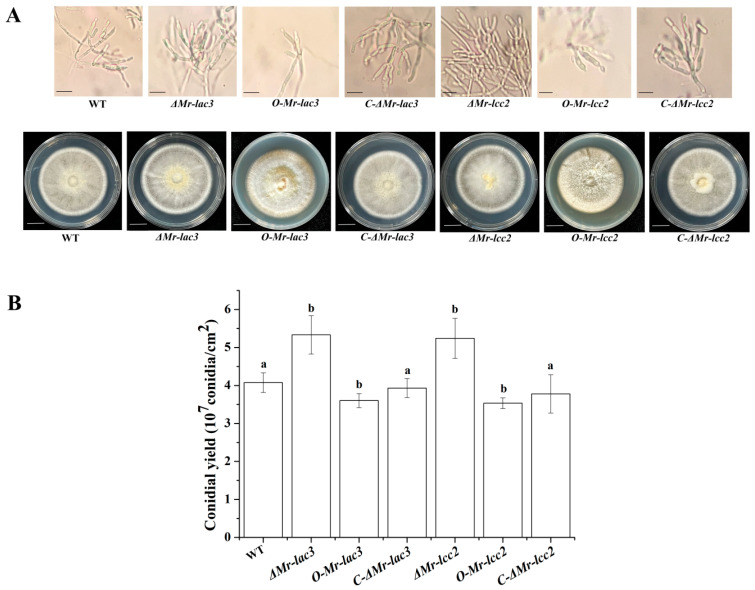
Conidiophores and conidial yields of the disruption, overexpression, and complementation strains of *Mr-lac3* and *Mr-lcc2*. (**A**). Top panel of the upper picture: Conidiophores were observed at 3 days post inoculation by spraying 100 µL of a conidial suspension (1 × 10^7^ conidia mL^−1^) on the PDA plate. Bottom panel: Colony morphology of the WT, disruption, overexpression, and complementation strains. Colony pictures were taken at 15 days post inoculation by applying 5 µL of a conidial suspension (1 × 10^7^ conidia mL^−1^) onto the center of a PDA plate (diameter 9 cm). Bar, 1 cm. (**B**). Conidial yields of the WT, disruption, overexpression, and complementation strains. Conidial yields were measured at 15 days post inoculation by spraying 100 µL of a conidial suspension (1 × 10^7^ conidia mL^−1^) onto the PDA plate. Values with different letters are significantly different (*p* < 0.05). Conidial yields were repeated three times with three PDA plates (diameter 9 cm) per repeat.

The phialides and conidiophores formed by *ΔMr-lac3* and *ΔMr-lcc2* were highly branched and formed densely clustered phialides where the conidia are produced. The conidial yields of *ΔMr-lac3* (5.33 × 10^7^) and *ΔMr-lcc2* (5.24 × 10^7^) were significantly higher than that of WT (4.08 × 10^7^). However, the overexpression strains had fewer branches and scattered phialides, with conidial yields of *O-Mr-lac3* (3.6 × 10^7^) and *O-Mr-lcc2* (3.54 × 10^7^) significantly lower than that of WT (Figure 1A,B).

### 3.3. Expression Patterns of Mr-lac3 and Mr-lcc2 in M. robertsii

**Figure 2:**
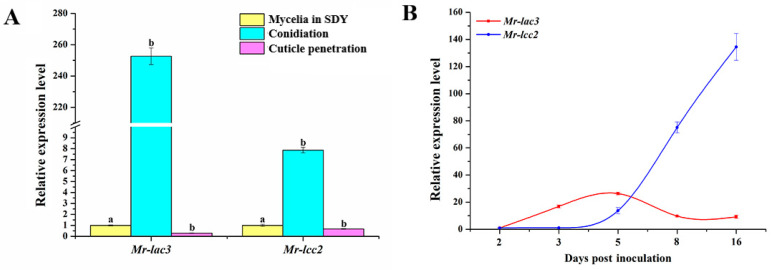
Expression patterns of *Mr-lac3* and *Mr-lcc2* in *M. robertsii*. (**A**). qRT-PCR analysis of *Mr-lac3* and *Mr-lcc2* expression patterns during mycelia grown in nutrient-rich SDY (Sabouraud dextrose broth plus 1% Yeast Extract), conidiation (5 days post inoculation by spraying 100 µL of 1 × 10^7^ conidia mL^−1^ conidial suspension onto the PDA plate), and cuticle penetration (appressoria-forming germlings on *Galleria mellonella* cuticle) in the wild-type strain (WT). The expression level of a gene during conidiation and cuticle penetration was calculated relative to that in mycelia grown in SDY, which was set to 1. Values with different letters are significantly different (*p* < 0.05). (**B**). A time course analysis of the expression of *Mr-lac3* and *Mr-lcc2* during conidiation in the WT strain. RNA was extracted at the 5 conidiation stages, 2, 3, 5, 8, and 16 days post inoculation, by spraying 100 µL of a conidial suspension (1 × 10^7^ conidia mL^−1^) onto the PDA plate. The expression level at day 2 was set to 1. qRT-PCR analyses were repeated three times.

Using qRT-PCR, we observed the expression patterns of *Mr-lac3* and *Mr-lcc2* in the wild-type (WT) strain during mycelia growth in nutrient-rich SDY, conidiation, and cuticle penetration. The gene expression of *Mr-lac3* and *Mr-lcc2* during the conidiation was, respectively, 250- and 7.5-fold greater than that during mycelia growth in SDY and cuticle penetration (Figure 2A) (*p* < 0.05), suggesting that *Mr-lac3* and *Mr-lcc2* are involved in conidiation. Further qRT-PCR analysis was conducted to examine the expression levels of *Mr-lac3* and *Mr-lcc2* in the five conidiation stages. The results showed that *Mr-lac3* reached its highest level at 5 days post inoculation (conidiation), remaining fairly constant until 16 days post inoculation (conidia maturation). On the other hand, *Mr-lcc2* increased gradually from 5 days to 16 days post inoculation (Figure 2B).

### 3.4. Mr-lac3 and Mr-lcc2 Negatively Regulated Conidiation

**Figure 3:**
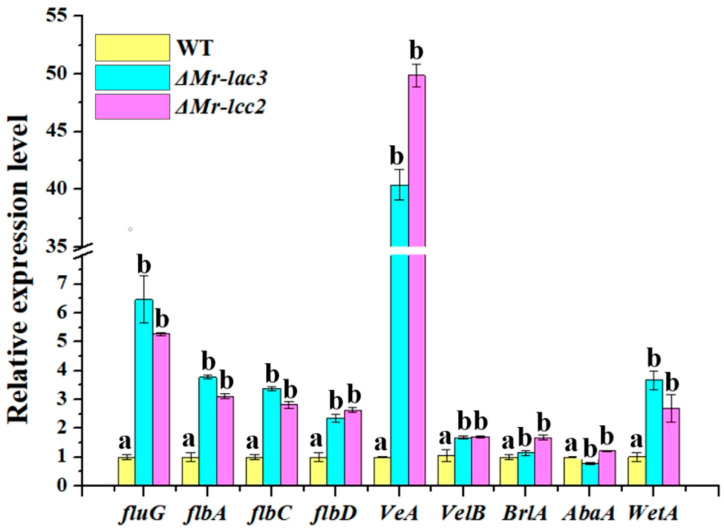
Mr-lac3 and Mr-lcc2 negatively regulated conidiation. RNA was extracted at 5 days post inoculation. Values with different letters are significantly different (*p* < 0.05). For each gene, the expression level in the WT strain was set to 1. qRT-PCR analyses were repeated three times with three replicates per repeat.

Previous studies have suggested that the formation and development of conidia were controlled by conserved regulatory pathways containing components such as *fluG*, *flbA*, *flbC*, *flbD*, *veA*, *velB*, *BrlA*, *AbaA*, and *WetA* in many filamentous fungi [25]. We conducted qRT-PCR to analyze whether there are regulatory relationships between these components of conidiation regulatory pathways and *Mr-lac3* and *Mr-lcc2*. In comparison to that in the WT strain, the relative expression levels of components in the conidiation regulatory pathways were significantly increased in *ΔMr-lac3* and *ΔMr-lcc2* (Figure 3). This suggests that *Mr-lac3* and *Mr-lcc2* may act as negative regulators of conidiation in *M. robertsii*.

### 3.5. Mr-lac3 and Mr-lcc2 Participated in Synthesizing Conidial Pigment

**Figure 4:**
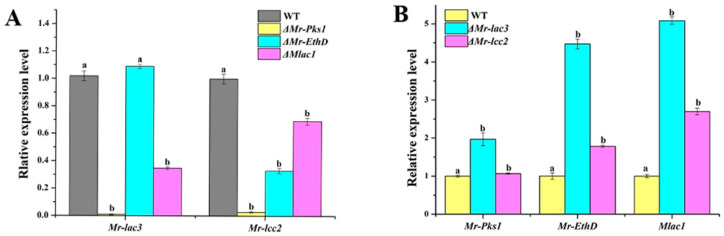
*Mr-lac3* and *Mr-lcc2* are involved in conidial pigment biosynthesis. (**A**). The expression levels of *Mr-lac3* and *Mr-lcc2* were compared between wild-type (WT) and knock-out mutants of the *Pks1* gene cluster. Values with different letters were found to be significantly different (*p* < 0.05). (**B**). The expression levels of *Mr-Pks1*, *Mr-EthD*, and *Mlac1* in the WT strain, *ΔMr-lac3*, and *ΔMr-lcc2*. RNA was extracted at 7 days post inoculation. Values with different letters are significantly different (*p* < 0.05). The expression level of each gene was set to 1 in the WT strain. For each gene, the expression level in the WT strain was set to 1. qRT-PCR analyses were repeated three times with three replicates per repeat.

In a previous study, a new pigmentation gene cluster was found in *M. robertsii*, which includes *Mr-Pks1*, *Mr-EthD*, and *Mlac1*. All three genes exercise feedback regulation of conidiation [18]. In this study, qRT-PCR revealed that the expression levels of *Mr-lac3* in *ΔMr-Pks1* and *ΔMlac1* are both significantly decreased compared with that in the WT strain, and those of *Mr-lcc2* were also significantly decreased in all three mutants of the pigmentation genes (Figure 4A). Conversely, the expression levels of the three pigmentation genes were all significantly increased in *ΔMr-lac3* and *ΔMr-lcc2* compared with that in the WT strain (Figure 4B). These findings suggest that *Mr-lac3* and *Mr-lcc2* are not only related to conidiation, but may also contribute to pigmentation.

### 3.6. Mr-lac3 and Mr-lcc2 Affect Cell Wall Integrity

**Figure 5:**
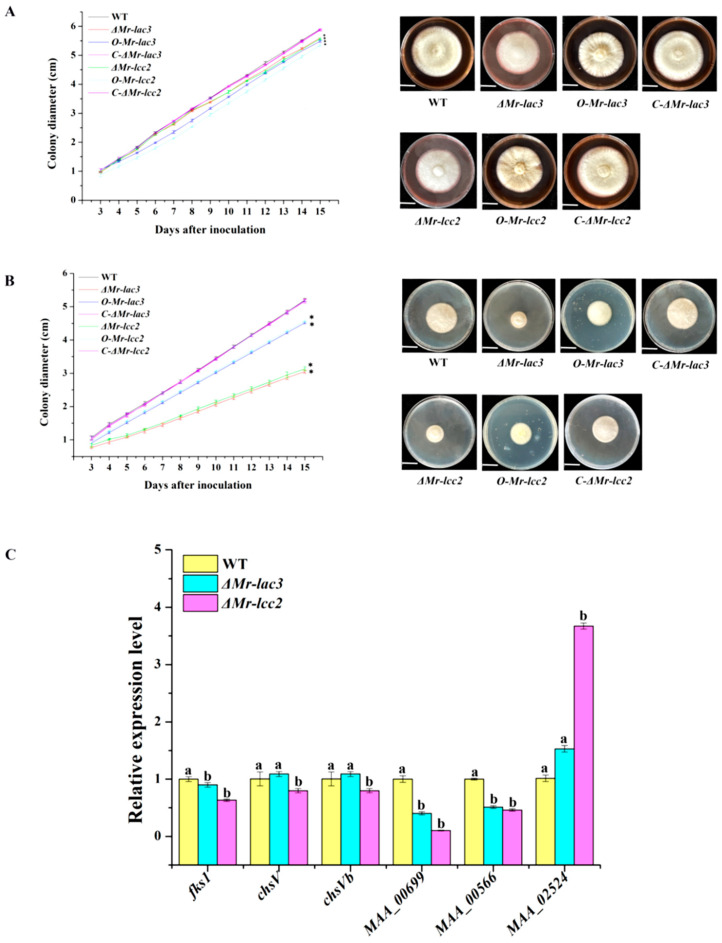
*Mr-lac3* and *Mr-lcc2* are involved in cell wall integrity. (**A**). The growth rate of colonies of knock-out (KO) mutants, overexpressed strains, and complementary strains of *Mr-lac3* and *Mr-lcc2* under abiotic stress treatments with Congo red (1.5 mg/mL). A 5 μL conidial suspension (1 × 10^7^ conidia mL^−1^) was inoculated in the center of the PDA containing Congo red (1.5 mg/mL) and cultured at 26 °C for a period of time. The diameter of the colonies was measured every day starting from the third day after inoculation. Pictures of the colonies were taken at 10 days post inoculation. (**B**). The growth rate of colonies of knock-out (KO) mutants, overexpressed strains, and complementary strains of *Mr-lac3* and *Mr-lcc2* under abiotic stress treatments with guaiacol (0.04%). A 5 μL conidial suspension (1 × 10^7^ conidia mL^−1^) was inoculated in the center of the PDA with guaiacol (0.04%) and cultured at 26 °C for a period of time. The diameter of the colonies was measured every day starting from 3 days after inoculation. Colony pictures were taken at 10 days post inoculation. Note: * *p* < 0.05. (**C**). The expression levels of components involved in cell integrity in *ΔMr-lac3* and *ΔMr-lcc2*. RNA was extracted at 5 days post inoculation. Values with different letters are significantly different (*p* < 0.05). For each gene, the expression level in WT was set to 1. qRT-PCR analyses were repeated three times with three replicates per repeat.

The integrity of the conidial cell wall reflects the quality of conidial development [26]. We determined the growth rates of *ΔMr-lac3* and *ΔMr-lcc2* on PDA plates containing the cell wall-disturbing agents Congo red (1.5 mg mL^−1^) and guaiacol (0.04%). They showed that the growth of both *ΔMr-lac3* and *ΔMr-lcc2* were significantly inhibited when compared with the WT strain, which were rescued in the corresponding complementation and overexpression strains (Figure 5 and Table S2). We used qRT-PCR to analyze the expression levels of several genes related to cell wall integrity, such as *fks1*, *chsV*, *chsVb*, *MAA_00699* (cell wall galactomanno protein), *MAA_00566* (Quinone oxidoreductase), and *MAA_02524* (cell wall biogenesis protein) in the *ΔMr-lac3* and *ΔMr-lcc2* during conidiation. Our results showed that compared to that in the wild-type (WT) strain, the expression levels of *fks1*, *MAA_00699*, and *MAA_00566* were significantly decreased in both *ΔMr-lac3* and *ΔMr-lcc2*. On the other hand, the expression levels of *chsV* and *chsVb* increased in *ΔMr-lac3* but decreased in *ΔMr-lcc2*, while the expression levels of *MAA_02524* increased in both mutants. Overall, our findings suggest that *Mr-lac3* and *Mr-lcc2* play roles in maintaining cell wall integrity.

### 3.7. Mr-lac3 and Mr-lcc2 Are Involved in Conidial Trehalose Synthesis

**Figure 6:**
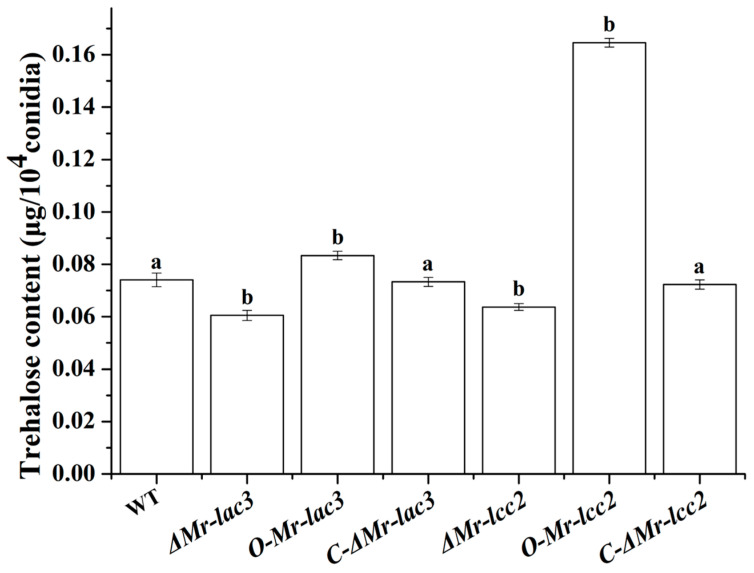
*Mr-lac3* and *Mr-lcc2* are involved in conidial trehalose synthesis. The 15-day-old conidia (2 × 10^8^ conidia) were washed with ddH_2_O three times, resuspended in 200 µL ddH_2_O, and incubated at 100 °C for 20 min. The suspension was then centrifuged for 10 min at 11,000× *g*, and the supernatant containing trehalose was collected. The amount of glucose liberated by the activity of trehalose was assayed using a glucose (GO) assay kit and converted into trehalose per conidium (measured in triplicate). Each sample without trehalose treatment served as a negative control. Note: There are significant differences between different letters (*p* < 0.05).

Trehalose, as a protective sugar, plays an important role in the synthesis and regulation of cell wall components and promotes the formation and development of fungal conidia. The trehalose content of the *Mr-lac3* and *Mr-lcc2* deletion, overexpression, and complementation strains was determined using anthrone colorimetry. The results showed that the trehalose levels in the *ΔMr-lac3* and *ΔMr-lcc2* strains were significantly lower than those of the wild type (WT), while the overexpression strains *O-Mr-lac3* and *O-Mr-lcc2* had significantly higher trehalose content compared to the WT (Figure 6). These findings suggested that *Mr-lac3* and *Mr-lcc2* are involved in the synthesis of conidial trehalose.

### 3.8. Tolerance of ΔMr-lac3 and ΔMr-lcc2 to Abiotic Stresses

**Figure 7:**
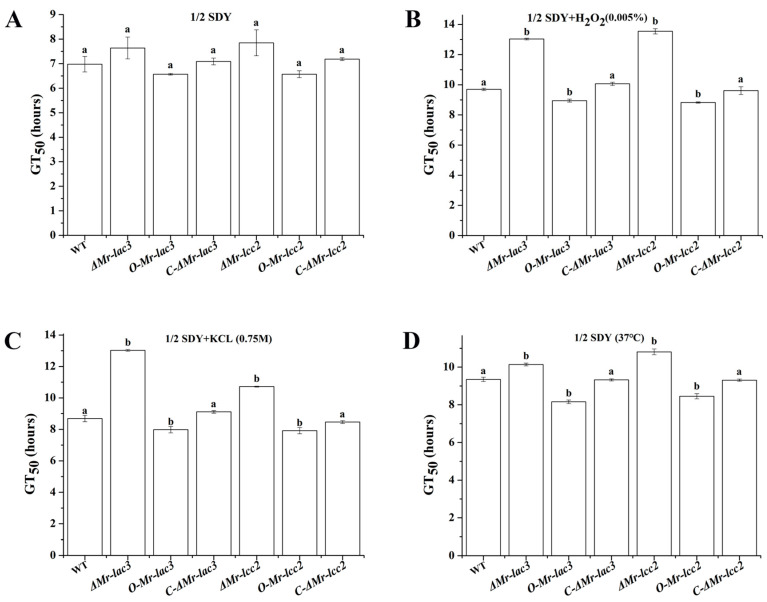
GT_50_ (time taken for 50% of conidia to germinate) values of knock-out (KO) mutants, overexpressed strains, and complementary strains of *Mr-lac3* and *Mr-lcc2* under optimal conditions (**A**) and three abiotic stress treatments [0.005% H_2_O_2_ (**B**), 0.75 M KCl (**C**), 37 °C (**D**)]. A 60 μL conidial suspension (4 × 10^7^ conidia mL^−1^) was inoculated into 3 mL of 1/2 SDY liquid medium and 1/2 SDY liquid medium with 0.005% H_2_O_2_ and 0.75 M KCL added in a Petri dish with a diameter of 3 cm. The culture was incubated at 37 °C and 26 °C for a specific period of time. Conidial germination was observed and counted under an inverted microscope every 2 h. Within the same abiotic stress treatment, values with different letters were significantly different (*p* < 0.05). The assays were repeated three times with three Petri dishes per repeat.

Trehalose is an important protective sugar that can enhance fungal tolerance to abiotic stress. Under optimal conditions (26 °C in 1/2 SDY medium), the deletion and overexpression of *Mr-lac3* and *Mr-lcc2* had no impact on the conidial germination of *M. robertsii*, as indicated by the GT_50_ (time taken for 50% of the conidia to germinate) (Figure 7). However, under oxidative stress (1/2 SDY supplemented with 0.005% H_2_O_2_), hyperosmotic stress (1/2 SDY supplemented with 0.75 M KCl), and heat stress (37 °C in 1/2 SDY), the *ΔMr-lac3* and *ΔMr-lcc2* germinated significantly slower than the WT. In contrast, the *O-Mr-lac3* and *O-Mr-lcc2* germinated significantly faster than the WT (*p* < 0.05) (Figure 7 and Table 1). In terms of growth rates on PDA plates, there was no significant difference at 37 °C and on plates containing 0.005% H_2_O_2_ or 0.75M KCl at 26 °C. Although the growth rate of the *O-Mr-lac3* was not significantly different from that of the WT under oxidative stress and heat stress, the growth of the strain under hyperosmotic stress was significantly faster than that of the WT. Similarly, the growth rate of the *O-Mr-lcc2* was not significantly different from that of the WT under oxidative stress, but the growth of the strain under hyperosmotic and heat stress was significantly faster than that of the WT (Figure S2 and Table S2).

### 3.9. Roles of Mr-lac3 and Mr-lcc2 in Pathogenicity

**Figure 8:**
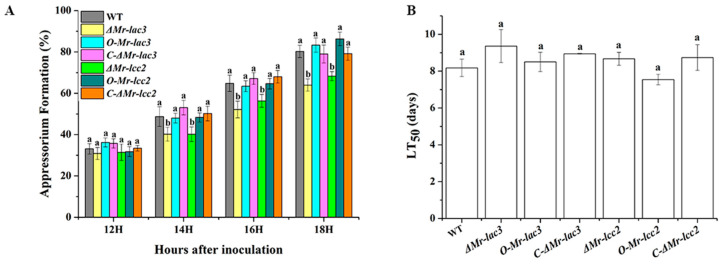
Pathogenicity of knock-out (KO) mutants, overexpressed strains, and complementary strains of *Mr-lac3* and *Mr-lcc2*. (**A**). The percentage of appressoria-forming germlings on a hydrophobic plastic surface. At each time point, values with different letters are significantly different (*p* < 0.05). Appressorium formation assays were repeated three times with three hydrophobic Petri dishes per repeat. Values with the same letter are not significantly different (*p* > 0.05). (**B**). LT_50_: time needed for 50% of a lethal dose. Pathogenicity assays were repeated three times with 40 insects of *Galleria mellonella* per repeat.

To investigate the roles of *Mr-lac3* and *Mr-lcc2* in pathogenicity, we assessed the appressoria formation rates on a hydrophobic surface and the abilities to infect the insect by cuticle penetration of *Mr-lac3* and *Mr-lcc2* knock-out mutants and overexpression strains. Our results indicated that the appressoria formation of *ΔMr-lac3* and *ΔMr-lcc2* were significantly delayed compared to that of the wild type (WT), but there was no significant difference in their LT_50_ values (the time taken to kill 50% of insects). In contrast, the appressoria formation rates and LT_50_ of *O-Mr-lac3* and *O-Mr-lcc2* did not differ significantly from the WT (Figure 8A,B). These findings suggested that *Mr-lac3* and *Mr-lcc2* play a role in the normal formation of appressoria.

### 3.10. A Schematic Model of Function and the Regulation of Mr-lac3 and Mr-lcc2

**Figure 9:**
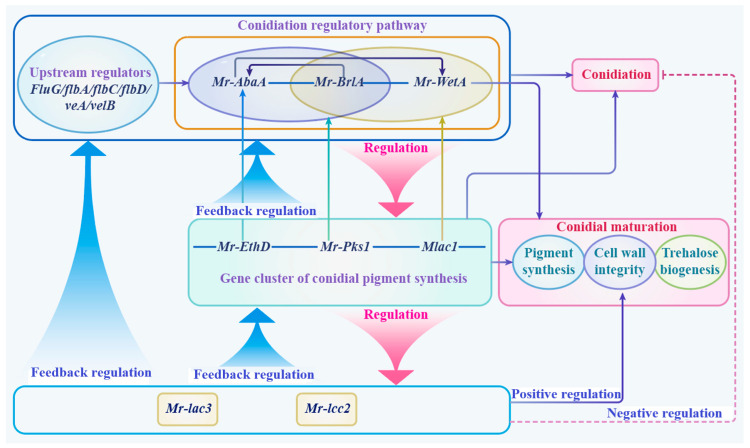
A schematic model of illustrating the function and regulation of *Mr-lac3* and *Mr-lcc2*.

In conclusion, we developed a schematic model illustrating the function and regulation of *Mr-lac3* and *Mr-lcc2* (Figure 9). *Mr-lac3* and *Mr-lcc2* were regulated by the conidiation regulatory pathway and the gene cluster of conidial pigment synthesis. In contrast, *Mr-lac3* and *Mr-lcc2* play a role in feedback regulation in these pathways. The regulatory mechanisms negatively influence conidiation while positively contributing to conidial maturation, which encompasses processes such as pigment synthesis, cell wall integrity, and trehalose biogenesis. Collectively, these processes enhance tolerance to abiotic stresses and contribute to pathogenicity.

## 4. Discussion

In this study, we identified two laccase genes, *Mr-lac3* and *Mr-lcc2,* in *M. robertsii* that contribute to conidiation, pigmentation, trehalose biosynthesis, cell wall integrity, and stresses tolerance.

Conidia, as propagules, have a highly conserved molecular mechanism of formation and development. In many ascomycete fungi, the conserved conidiation regulatory pathway containing *BrlA*, *AbaA*, and *WetA* was found, which sequentially regulates conidia generation and development [18,27,28,29,30]. In *M. robertsii*, *Mr-WetA* plays key roles in conidiation and conidial maturation. Previously, we found that *ΔMr-WetA* produced red and distorted conidia with a significant decrease in conidial yield [18]. And by the transcriptomic analysis of the *ΔMr-WetA* during conidiation, we found significant changes in the expression levels of many genes involved conidiation, pigment synthesis, cell wall integrity, and so on. Among these, we identified a significant down-regulation of the expression levels of two laccases encoding genes *Mr-lac3* and *Mr-lcc2*, suggesting that the Mr-WetA may control conidiogenesis and conidium maturation through the regulation of *Mr-lac3* and *Mr-lcc2*. We constructed the knock-out mutants and overexpression strains of *Mr-lac3* and *Mr-lcc2*; the *ΔMr-lac3* and *ΔMr-lcc2* showed significant increases in conidial yields, while those of *O-Mr-lac3* and *O-Mr-lcc2* decreased significantly. QRT-PCR analysis showed that *Mr-lac3* and *Mr-lcc2* are highly expressed during the conidiating stage. And the expression levels of activators (*fluG*, *flbA*, *flbC*, *flbD*, *VeA*, and *VelB*) in the conidiation pathway and components of the conserved conidiation regulatory pathway (*Mr-BrlA*, *Mr-AbaA*, and *Mr-WetA*) were significantly up-regulated in *ΔMr-lac3* and *ΔMr-lcc2* compared to the WT. This suggests that *Mr-lac3* and *Mr-lcc2* may play a negative regulatory role in conidial formation, but they may not be regulated by Mr-WetA during this process.

The synthesis of fungal conidial pigments is a very important process during conidial development, and different pigments may play roles at different stages of conidial development [31]. In the early stages, the synthesis of some pigments can promote the formation and strengthening of the cell wall, and many pigments can help conidia resist abiotic stresses, enhancing the survival rate [32]. In previous studies, we discovered a new gene cluster in *M. robertsii* responsible for pigment synthesis. This cluster includes the genes *Mr-Pks1*, *Mr-EthD*, and *Mlac1*, which produce pigments that help the *M. robertsii* withstand UV radiation and other stressors [18]. In our current study, we observed a significant decrease in the expression levels of *Mr-lac3* and *Mr-lcc2* in three knock-out mutants of the pigment synthesis gene cluster, compared to the wild-type (WT) strain. Conversely, the expression levels of the three genes in the pigment synthesis gene cluster were notably increased in the *ΔMr-lac3* and *ΔMr-lcc2*. This suggests that *Mr-lac3* and *Mr-lcc2* are positively regulated by the pigment synthesis gene cluster and, in turn, provide them with feedback regulation. And although the color of conidia in the *ΔMr-lac3* and *ΔMr-lcc2* did not change directly, they may also be involved in regulating pigment synthesis.

The integrity of the conidial cell wall reflects the quality of conidia in development [26]. *ΔMr-lac3* and *ΔMr-lcc2* inoculated on PDA plates with different cell wall disruptors such as Congo red and guaiacol showed significantly slower growth rates compared to the wild type. Further qRT-PCR analysis revealed significant changes in the expression levels of several genes related to cell wall integrity during conidiation in the *ΔMr-lac3* and *ΔMr-lcc2*, indicating the involvement of *Mr-lac3* and *Mr-lcc2* in maintaining cell wall integrity. Trehalose participates in the synthesis of conidial cell wall polysaccharides, enhancing mechanical strength and stability [33]. Our analysis revealed notable decreases in conidial trehalose contents in *ΔMr-lac3* and *ΔMr-lcc2*. Conversely, the overexpression strains of *O-Mr-lac3* and *O-Mr-lcc2* both significantly increased in trehalose content. This indicated that *Mr-lac3* and *Mr-lcc2* play a role in the synthesis of trehalose in conidial cell walls, which is crucial for maintaining cell wall integrity.

Pigments and trehalose are essential for conidial development, helping fungi tolerate different abiotic stresses [34]. We found that the conidial germination rates of the *ΔMr-lac3* and *ΔMr-lcc2* did not show significant differences compared to the wild type under optimal growth conditions, but significantly decreased under oxidative, highly osmotic, and heat stress conditions; conversely, the conidial germination rates of the *O-Mr-lac3* and *O-Mr-lcc2* were significantly accelerated. Additionally, when measuring the colony diameter on PDA plates, we found that the growth rates of the *ΔMr-lac3* and *ΔMr-lcc2* were not significantly different from the wild type under various conditions, such as optimal, high-temperature, oxidative, and highly osmotic conditions. However, the growth rate of *O-Mr-lac3* was notably faster only under high osmotic stress, while that of *O-Mr-lcc2* was significantly accelerated under high osmotic and heat stress. Knocking out *Mr-lac3* and *Mr-lcc2* weakened the resistance of *M. robertsii* to abiotic stresses, whereas overexpressing them enhanced it’s tolerance to some extent. This suggests that *Mr-lac3* and *Mr-lcc2* may play a role in maintaining normal conidial development and stress tolerance by participating in the synthesis of conidial pigments and trehalose.

Conidia play a crucial role as the propagules in entomopathogenic fungi, enabling them to initiate infection and spread virulence [35]. Our analysis showed that the appressorial formation of the *ΔMr-lac3* and *ΔMr-lcc2* were significantly delayed compared to the wild-type strain. Additionally, the virulence of insect infection by cuticle penetration (LT_50_) decreased, although there was no significant difference compared to the wild-type strain. This indicated that knocking out *Mr-lac3* and *Mr-lcc2* affects the conidial development of *M. robertsii*, thereby influencing its pathogenicity to a certain extent.

## Figures and Tables

**Table 1:** Relative inhibition of germination rates of knock-out (KO) mutants, overexpressed strains, and complementary strains of *Mr-lac3* and *Mr-lcc2* under three abiotic stresses. Within the same abiotic stress treatment, values appended by different letters were significantly different (*p* < 0.05). The assays were repeated three times.

Strains	Relative Germination Inhibition		
	H_2_O_2_ Stress	KCL Stress	Heat Stress
WT	0.39 ± 0.01 ^a^	0.25 ± 0.03 ^a^	0.34 ± 0.02 ^a^
*ΔMr-lac3*	0.71 ± 0.01 ^b^	0.71 ± 0.01 ^b^	0.33 ± 0.01 ^a^
*O-Mr-lac3*	0.36 ± 0.02 ^b^	0.22 ± 0.03 ^b^	0.24 ± 0.01 ^b^
*C-ΔMr-lac3*	0.40 ± 0.01 ^a^	0.27 ± 0.01 ^a^	0.32 ± 0.01 ^a^
*ΔMr-lcc2*	0.73 ± 0.02 ^b^	0.37 ± 0.01 ^b^	0.38 ± 0.02 ^b^
*O-Mr-lcc2*	0.34 ± 0.01 ^b^	0.21 ± 0.03 ^b^	0.29 ± 0.02 ^b^
*C-ΔMr-lcc2*	0.37 ± 0.01 ^a^	0.26 ± 0.01 ^a^	0.30 ± 0.01 ^a^

## Data Availability

The original contributions presented in this study are included in the article/Supplementary Materials. Further inquiries can be directed to the corresponding author.

## Supplementary Materials

The following supporting information can be downloaded at: https://www.mdpi.com/article/10.3390/jof11030176/s1, Figure S1: Construction of knock-out (KO) mutants, overexpressed strains and complementary strains of *Mr-lac3* and *Mr-lcc2* in *Metarhizium*.; Figure S2: The growth rate of colonies of knock-out (KO) mutants, overexpressed strains and complementary strains of *Mr-lac3* and *Mr-lcc2* under optimal conditions and three abiotic streses treatments (0.005%H_2_O_2_, 0.75M KCL and 37 °C).; Table S1: Primers used in the article.; Table S2: The relative inhibitory growth rate of colonies of knock-out (KO) mutants, overexpressed strains and complementary strains of *Mr-lac3* and *Mr-lcc2* under optimal conditions and three abiotic streses treatments (0.005% H_2_O_2_, 0.75M KCL, 37 °C, 1.5mg/mL Congo red, 0.04% Guaiacol).
