# Supplementary material for: Mr-lac3 and Mr-lcc2 in Metarhizium robertsii Regulate Conidiation and Maturation, Enhancing Tolerance to Abiotic Stresses and Pathogenicity
Source: J Fungi (Basel). 2025 Feb 22;11(3):176. doi: 10.3390/jof11030176 (PMC11942773; doi:10.3390/jof11030176)
Supplement: Supplementary file 1 [file jof-11-00176-s001.zip › jof-3454687-supplementary.pdf]

Table S1. Primers used in the article.

Primer	Sequence	Usage
Bar-up	CGCCTGGACGACTAAACC	
Bar-down	CGCCTGGACGACTAAACC	Confirmation of the Disruptions
ΔMr-lac3-5-1	GCTCTAGAGCTACTGTCAGTGAATG	Disruption of *Mr-lac3*
ΔMr-lac3-5-2	CGGAATTCTGACCTCATACGTGAAG	
ΔMr-lac3-3-1	GGACTAGTGCTCCTGATTCTATGAG	
ΔMr-lac3-3-2	GGTTTAAACATGAAACCGCAGAGTG	
ΔMr-lac3-CF-1	CAGATAAACCAACATCG	Confirmation of the Disruption
ΔMr-lac3-CF-2	TGCAACATCTCACCTTG	of Mr-lac3
Mr-lac3-5	ATTGCATGCTCTCACACTAGTCATCCGTCTTTAGCATC	Cloning the genomic clone of
Mr-lac3-3	ACCGACGGAATTGAGGATATCATCTCATACCGTCACAC	*Mr-lac3* for complementation
Mr-lac3-ORF-5	ATTGCATGCTCTCACACTAGTCATGCCACTTGTGTTAC	Conformation of the
Mr-lac3-ORF-3	ACCGACGGAATTGAGGATATCAGGAAGGCAGACAGATC	conplementation of *Mr-lac3*
O-Mr-lac3-5	CGGGATCCACGAGTTTGACCTCATC	Cloning the genomic clone of
O-Mr-lac3-3	CGGAATTCCTCATAGAATCAGGAGC	*Mr-lac3* for overexpression
ΔMr-lcc2-5-1	GGACTAGTGCATGCGTTAGTAGTTC	Disruption of *Mr-lac3*
ΔMr-lcc2-5-2	CGGAATTCTCTATCGTGTCTCTCAC	
ΔMr-lcc2-3-1	GGACTAGTTCTGTAGGTTGCCTATC	
ΔMr-lcc2-3-2	GGGATATCTGTATGGTAATGCCGTC	
ΔMr-lcc2-CF-1	GCGAATAAACACGAGTC	Conformation of the disruption
ΔMr-lcc2-CF-2	GAATCTCAGTCCATGTC	of Mr-lcc2
Mr-lcc2-5	GCTCTAGACATGCCACTTGTGTTAC	Cloning the genomic clone of
Mr-lcc2-3	CGGATATCAGGAAGGCAGACAGATC	*Mr-lcc2* for complementation
Mr-lcc2-ORF-5	CGGATATCATGACTTTAATCGAGCG	Conformation of the
Mr-lcc2-ORF-3	CGGATATCTTAAACACCGGAATCGT	conplementation of *Mr-lcc2*
O-Mr-lcc2-5	CGGATATCCATTACAGGCGATACGC	Cloning the genomic clone of
O-Mr-lcc2-3	CGGATATCCAGGACCTAATGCAGGC	*Mr-lcc2* for overexpression
Mr-lac3-RT-5	TGATAGAGCGTGTTTGGGAGATGG	
Mr-lac3-RT-3	TGCTTGGTTGACAGGAGTTACAGG	qRT-PCR for *MAA_lac3*
Mr-lcc2-RT-5	ACCGCATCGTAGATCCACTTACAC	
Mr-lcc2-RT-3	TGAGGTTCAGGTTATCTGGCTTCC	qRT-PCR for *MAA_lcc2*
Mr-BrlA-RT-5	ACCTGGCATCAAGACACTCC	
Mr-BrlA-RT-3	GATCCGGATAGGCTGTGTG	qRT-PCR for *MAA_Br1A*
Mr-AbaA-RT-5	TTTGATGCTCGTAGTGCCAG	
Mr-AbaA-RT-3	GGTATCCACGCCAGAGTTGT	qRT-PCR for *MAA_AbaA*
Mr-WetA-RT-5	TCTCCAAAACCTCCCAACAC	
Mr-WetA-RT-3	CTGCTTTCCTATTTCGTCGC	qRT-PCR for *MAA_WetA*
Mr-fluG-RT-5	TGCGGGTTGAATACGG	
Mr-fluG-RT-3	CTCCACCTCTTTCTCCTTGA	qRT-PCR for *MAA_fluG*
Mr-flbA-RT-5	ACTCCAAAGGGCATCACG	
Mr-flbA-RT-3	CAACAAAGCGGCGGAATA	qRT-PCR for *MAA_flbA*
Mr-flbC-RT-5	TTTCCAATCTACGACGACA	
Mr-flbC-RT-3	AGTCCGATTGATGGTCTTC	qRT-PCR for *MAA_flbC*

Continued table S1

Primer	Sequence	Usage
Mr-flbD-RT-5	AACGATGGGCTGAGATTG	
Mr-flbD-RT-3	GGTGATTGAGTTTCGGATG	qRT-PCR for *MAA_flbD*
Mr-veA-RT-5	TGAAAGCCAATAGCGACAGAC	
Mr-veA-RT-3	CGGGAAAGATGAAGTAACCAG	qRT-PCR for *MAA_veA*
Mr-velB-RT-5	CAGGGAAACCTCTTGGGA	
Mr-velB-RT-3	TCGCCGAAACCGCACATT	qRT-PCR for *MAA_velB*
Mr-Pks1-RT-5	AACTACTTTGGAGACGGCCA	
Mr-Pks1-RT-3	ACCAAGAGGCCAACTTTGAG	qRT-PCR for *MAA_Pks1*
Mr-EthD-RT-5	CAACATGCTTCTTACACCGC	
Mr-EthD-RT-3	TCATAGTCGGACAGCTTGGA	qRT-PCR for *MAA_EthD*
Mlac1-RT-5	GGGCCTCCTCGTATTTTGTC	
Mlac1-RT-5	GAAAGCGTCCTCAACCAGAC	qRT-PCR for *Mlac1*
Mr-fks1-RT-5	TACTACAATGCCGATCCGAA	
Mr-fks1-RT-3	TCAGAATCCTCCTCAGACCC	qRT-PCR for *fks1*
Mr-chsV-RT-5	CCGTTGTATTTCTTGGTGA	
Mr-chsV-RT-3	TGTGGCTGAGGTATCGTAT	qRT-PCR for *chsV*
Mr-chsVb-RT-5	CCGAAGACGGATGTGTGAT	
Mr-chsVb-RT-3	CGAGGTGTGGAAGGATTAT	qRT-PCR for *chsVb*
MAA_00699-RT-5	ATGAGAGTTGCGTACCCCTC	
MAA_00699-RT-3	CAACGACCATTGGCTTGACA	qRT-PCR for *MAA_00699*
MAA_00566-RT-5	AACGGCAATTGCAGCAATGT	
MAA_00566-RT-3	TACCACCTCGGACCTCAGAG	qRT-PCR for *MAA_00566*
MAA_02524-RT-5	TGTCTACTCCACCGAAGTGTCTAGC	
MAA_02524-RT-3	CTGTTCCCGTTGTCTGCATTGTATG	qRT-PCR for *MAA_02524*
Gpd-RT-5	GTCGTCATCTCTGCTCCCTC	
Gpd-RT-3	CAATGGTGAACTTGTCGTGG	qRT-PCR for reference
Tef-RT-5	AGGCTGACTGCGCTATTCTC	
Tef-RT-3	ACTTGGTGGTGTCCATCTTG	qRT-PCR for reference

Table S2. The relative inhibitory growth rate of colonies of knock-out (KO) mutants, overexpressed strains and complementary strains of *Mr-lac3* and *Mr-lcc2* under optimal conditions and three abiotic streses treatments (0.005% H_2O_2, 0.75M KCL, 37℃, 1.5mg/mL Congo red, 0.04% Guaiacol).

	H_2O_2 stress	KCL stress	Heat stress	Congo red stress	Guaiacol stress
WT	0.034 ± 0.008^{a}	0.710 ± 0.005^{a}	0.674 ± 0.001^{a}	0.182 ± 0.001^{a}	0.279 ± 0.007^{a}
ΔMr-lac3	0.063 ± 0.002^{a}	0.722 ± 0.004^{a}	0.685 ± 0.008^{a}	0.229 ± 0.001^{b}	0.580 ± 0.007^{b}
O-Mr-lac3	0.011 ± 0.007^{a}	0.683 ± 0.004^{b}	0.683 ± 0.009^{a}	0.190 ± 0.007^{a}	0.278 ± 0.004^{a}
C-ΔMr-lac3	0.048 ± 0.008^{a}	0.712 ± 0.005^{a}	0.680 ± 0.004^{a}	0.192 ± 0.004^{a}	0.291 ± 0.008^{a}
ΔMr-lcc2	0.043 ± 0.004^{a}	0.717 ± 0.004^{a}	0.675 ± 0.004^{a}	0.219 ± 0.004^{b}	0.560 ± 0.008^{b}
O-Mr-lcc2	0.079 ± 0.157^{a}	0.679 ± 0.017^{b}	0.656 ± 0.005^{b}	0.158 ± 0.008^{b}	0.268 ± 0.005^{a}
C-ΔMr-lcc2	0.050 ± 0.008^{a}	0.715 ± 0.004^{a}	0.683 ± 0.004^{a}	0.186 ± 0.004^{a}	0.290 ± 0.004^{a}

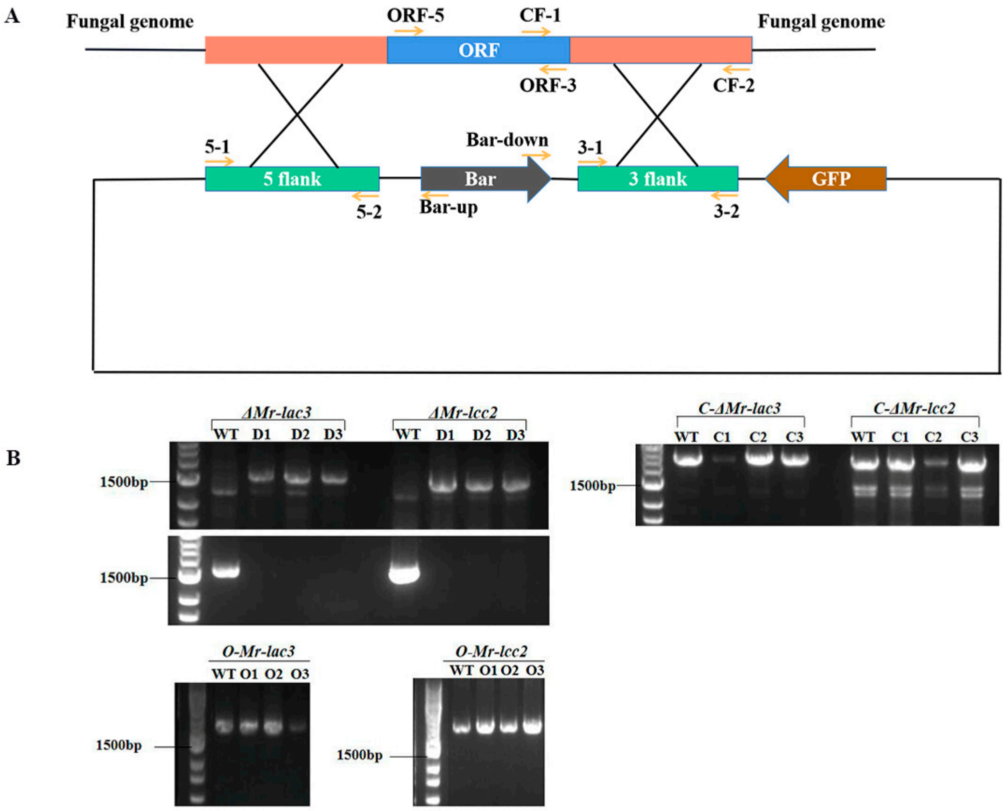

Figure S1. Construction of knock-out (KO) mutants, overexpressed strains and complementary strains of *Mr-lac3* and *Mr-lcc2* in *Metarhizium*. A. The disruption plasmid of a gene (bottom) and its relative position in the fungal genome (up). B. Confirmation of the gene disruption by PCR in the mutants with PPT resistance and without a GFP signal. In each gene, D1 to D3 designate three independent disruption mutants, and WT is the wild-type strain. Top panel of the upper picture: PCR conducted with the primers Bar-up and the confirmation primer CF2 of each gene, PCR products can be obtained only from the disruption mutants of the gene; bottom panel: PCR conducted using primers CF1 and CF2; PCR products can be obtained only in WT strain. Confirmation of the complementation of deletion mutants by PCR using the primers ORF-5 and ORF-3. C1, C2 and C3: independent complemented strains; WT: wild-type strain. Confirmation of the overexpressed strains by PCR using the primers ORF-5 and ORF-3. O1, O2 and O3: independent overexpressed strains; WT: wild-type strain. The positions of all primers are shown in A. The DNA ladder (DL 10004) on the left is purchased from Generay (Shanghai, China).

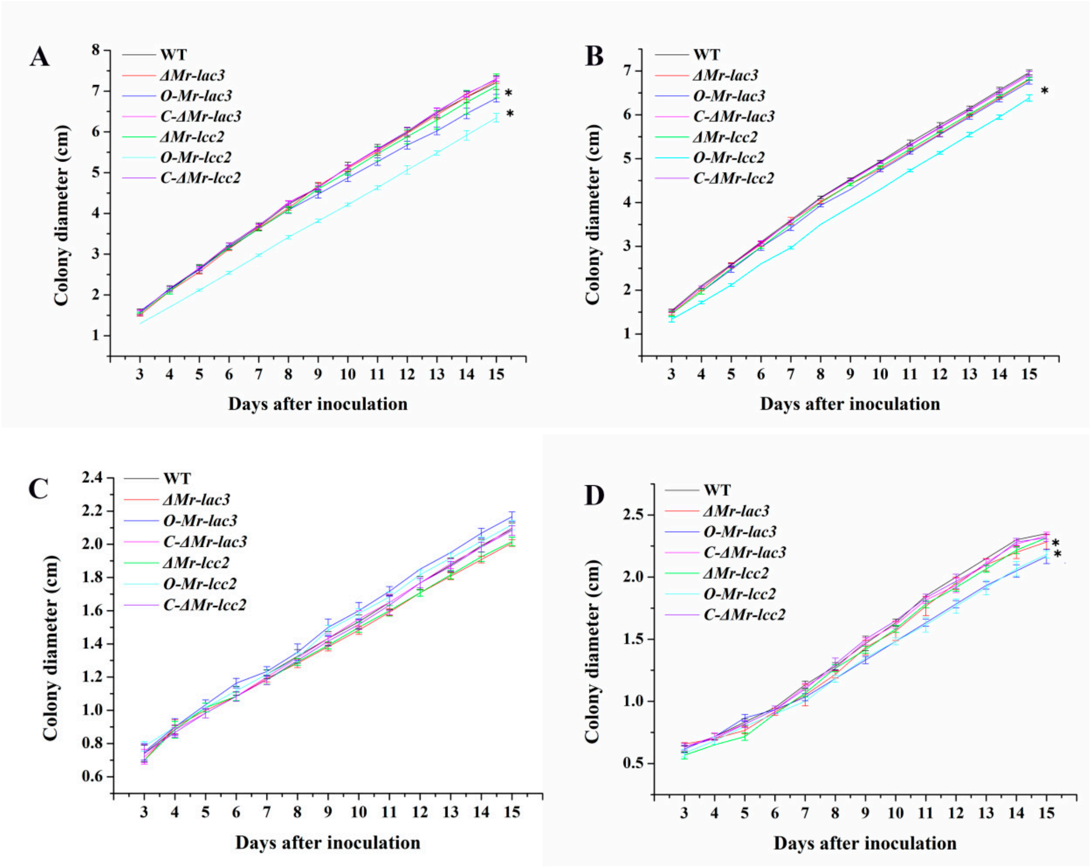

Figure S2. The growth rate of colonies of knock-out (KO) mutants, overexpressed strains and complementary strains of *Mr-lac3* and *Mr-lcc2* under optimal conditions and three abiotic streses treatments (0.005%H_2O_2, 0.75M KCL and 37℃). 5 μL conidial suspension (1×10^7 conidia mL^{-1}) was inoculated in the center of the PDA (A) and PDA supplemented with 0.005% H_2O_2 (B), 0.75M KCL (C) and incubated at 26℃ and 37℃ (D) for a period of time. The diameter of the colonies was measured every day from the 3 days after inoculation and then drawn growth curve. The experiment was repeated for 3 times, and 3 petri dishes were set for each repeat. Note: *$P < 0.05$.
